# Sempervirine Inhibits Proliferation and Promotes Apoptosis by Regulating Wnt/β-Catenin Pathway in Human Hepatocellular Carcinoma

**DOI:** 10.3389/fphar.2021.806091

**Published:** 2021-12-07

**Authors:** Rongcai Yue, Haiping Liu, Yaxin Huang, Jing Wang, Dongmei Shi, Yanping Su, Yufei Luo, Ping Cai, Guilin Jin, Changxi Yu

**Affiliations:** ^1^ School of Pharmacy, Fujian Medical University, Fuzhou, China; ^2^ Fujian Key Laboratory of Drug Target Discovery and Structural and Functional Research, Fujian Medical University, Fuzhou, China; ^3^ Fujian Provincial Key Laboratory of Environment Factors and Cancer, School of Public Health, Fujian Medical University, Fuzhou, China

**Keywords:** sempervirine, wnt/β-catenin, alkaloids, *Gelsemium elegans* benth., hepatocellular carcinoma

## Abstract

*Gelsemium elegans* (*G. elegans*) Benth., recognized as a toxic plant, has been used as traditional Chinese medicine for the treatment of neuropathic pain and cancer for many years. In the present study, we aim to obtain the anti-tumor effects of alkaloids of *G. elegans* and their active components in hepatocellular carcinoma (HCC) and the potential mechanism was also further investigated. We demonstrated that sempervirine induced HCC cells apoptosis and the apoptosis was associated with cell cycle arrest during the G_1_ phase, up-regulation of p53 and down-regulation of cyclin D1, cyclin B1 and CDK2. Furthermore, sempervirine inhibited HCC tumor growth and enhances the anti-tumor effect of sorafenib *in vivo*. In addition, inactivation of Wnt/β-catenin pathway was found to be involved in sempervirine-induced HCC proliferation. The present study demonstrated that alkaloids of *G. elegans* were a valuable source of active compounds with anti-tumor activity. Our findings justified that the active compound sempervirine inhibited proliferation and induced apoptosis in HCC by regulating Wnt/β-catenin pathway.

## Introduction

Hepatocellular carcinoma (HCC), the most common form of primary liver cancer, is the fourth leading cause of cancer-related death worldwide (Villanueva, 2019). Despite improvements in detection and clinical treatment strategies, the 5-years survival rate for HCC is less than 20% (Yu et al., 2021). Conventional chemotherapeutic and radio therapeutic treatments have caused serious problems, and resistance to chemotherapy is frequently observed (Craig et al., 2020). Sorafenib is recommended as the first-line treatment options for HCC patients, with primary concerns of high dose toxicity and limited anti-cancer effects (Wang et al., 2021). Therefore, developing novel efficient drugs and searching more effective therapies or synergistic agents are necessary for improving HCC therapy.

For over decades, a goal of clinical oncology has been the development of therapies promoting the effective elimination of cancer cells by apoptosis (Ichim and Tait, 2016; Carneiro and El-Deiry, 2020). In addition, the cell cycle is a complex and strictly controlled process, which is controlled by several cyclin dependent kinase (CDK) complexes. p53 is the most widely studied tumor suppressor and mediates a variety of anti-proliferative processes through cell cycle and apoptosis (Hu et al., 2021).

Wnt signaling pathway, as one of the important conserved pathways in mammal, is involved in the differentiation, development and formation of all human organs, hematopoietic system and reproductive system (Perugorria et al., 2019). Therefore, the disorder of Wnt pathway is closely related to a variety of diseases, including tumors (Wang et al., 2019; Parsons et al., 2021). In normal cells, the Wnt pathway switch is not turned on. At this time, the Wnt pathway activity remains at a low level, which is mainly controlled by β-catenin complex, which is presented the excess in the ubiquitinated cytoplasm to the proteasome for degradation, thus silencing the downstream target genes. When Wnt pathway is activated, the binding of upstream Wnt ligands and receptors leads to the disintegration of β-catenin complex, the accumulation of β-catenin in the cytoplasm, entering the nucleus and binding with transcription factor TCF to start the expression of downstream oncogenes, including c-Myc, cyclin D1 and surviving (Li et al., 2021). At present, several drugs targeting Wnt pathway are undergoing clinical trials and are expected to be on the market (Martin-Orozco et al., 2019; Ashrafizadeh et al., 2020). Hence, Wnt pathway inhibitors can not only inhibit the growth of HCC cells, but also reduce the drug resistance of cells and sensitize chemotherapeutic drugs.

Natural products from plants are potentially important resources in the context of drug discovery for cancer therapy (Greco et al., 2021). Importantly, increasing evidence has reported that apoptosis induction or suppression might be one of the predominant molecular mechanisms whereby natural products could be used to treat cancer (Carneiro and El-Deiry, 2020). In addition, lots of active natural products based on apoptosis regulation, such as taxol, camptothecin and curcumin (Ashrafizadeh et al., 2020), have been used as clinical drugs to treat cancer (Mullard, 2016). *Gelsemium elegans* Benth. (*G. elegans*), also known as heartbreaking grass, has been used as a traditional Chinese medicine for the treatment of neuropathic pain and cancer for many years (Wang et al., 2015). Studies on the chemical constituents of the genus plants show that all species are rich in alkaloids, especially indole alkaloids, including gelsdine, gelsmeine, humantenine, koumine, sarpagine and yohimbane (Jin et al., 2014). Among them, structurally representative alkaloids sempervirine, gelsemine, humantene, and koumine have possessed anti-tumor, analgesic, anti-inflammatory and immunomodulatory pharmacological activities (Zhang and Wang, 2015; Pan et al., 2016; Yue et al., 2019; Jin et al., 2021). In the present study, we demonstrated that sempervirine inhibited proliferation and promoted apoptosis by regulating Wnt/β-catenin pathway in HCC.

## Materials and Methods

### Reagents and Materials

DMEM culture medium and fetal bovine serum (FBS) were purchased from Gibco (Grand Island, NY). The doxorubicin (DOX, catalog #ST1285), CCK8 (catalog #C0043), apoptosis detection kit (catalog #C1062L), TOPFlash (catalog #D2501) and FOPFlash (catalog #D2503) was purchased from Beyotime (Shanghai, China). Anti-human β-catenin (catalog #8480), cyclin D1 (catalog #55506), cyclin B1 (catalog # 12,231), survivin (catalog # 2808), c-Myc (catalog #18583), CDK2 (catalog #18048), p53 (catalog #2527), β-actin (catalog #4970) and Histone H3 (catalog #4499) antibodies were bought from Cell Signaling Technology (Beverly, MA, United States). FH535 (catalog #HY-15721) and BML-284 (catalog #HY-19987) was purchased from Med Chem Express (MCE, United States). Humantenidine, gelsemine, koumine, gelsenicin, gelsevirine, and sempervirine (HPLC≥98%) were purchased from Shanghai Yuanye Bio-Technology Co., Ltd. The roots and stems of *G. elegans* were bought from a commercial source and authenticated by the Department of Pharmacognosy, School of Pharmacy, Fujian Medical University as previous reported (Liu Hao et al., 2008). Other chemicals were of analytical grade and commercially available.

### Extraction the Total Alkaloid and Isolation of Alkaloid Compounds

The total alkaloid was extracted as previously described (Su et al., 2011). Briefly, *G. elegans* dry powder was refluxed in 95% ethanol for 3 h and the extraction was repeated for three times. The extracts were combined, dissoluted by 2% hydrochloric acid, and extracted again for another three times. The pH was adjusted to 11 with 5 M NaOH prior to extraction of total alkaloids. At the same time, the total extract and non alkaloid fraction were also reserved for subsequent experiment to detect activity. Alkaloidal compounds were then successfully separated using pH-zone-refining counter-current chromatography and compared with commercial standards purchased from Shanghai yuanye Bio-Technology Co., Ltd.

### Cell Lines and Cell Culture

HepG2, Huh7 and the normal cell line LX-2 were obtained from GuangZhou Jennio Biotech Co.,Ltd. The cells were grown under standard conditions (37°C with 5% CO_2_) using DMEM medium supplemented with 10% FBS.

### Cell Viability Assay

Cell viability was determined by the CCK8 assay. Cells were treated with sempervirine (0, 0.1, 0.5, 1, 2.5, 5, and 10 μM) for 24, 48, and 72 h, respectively. 0.1 μM Doxorubicin was used as a positive control. The absorbance at 450 nm was read using a BioTek Synergy two plate reader (BioTek Instruments, Inc.).

### Cell Apoptosis Assay

After digestion with trypsin, the cells were collected, washed with PBS and resuspended by 1× Binding buffer. Annexin V-FITC and solution were added and react at room temperature for 15 min, then detected by flow cytometry (FACSCalibur, BD) within 1 h.

### Cell Cycle Assay

Cells were incubated with sempervirine (0.5 and 1 μM) for 24 h. After treatment, the cells were fixed with cold 75% ethanol overnight at 4°C. Then the cells were incubated with PI staining solution (50 μg/mL) and RNase A solution (100 μg/mL) and incubated for 30 min. Cell cycle distribution were analyzed by flow cytometry (FACSCalibur, BD).

### Luciferase Reporter Assay

Cells were co-transfected with TOPflash or FOPflash vector together with luciferase reporter vector pRL-TK as the control for transfection efficiency as previous reported (Cheng et al., 2017). After 12 h of transfection, cells were incubated with sempervirine for another 24 h. Luciferase activity was measured and normalized according to the recommended protocol (Promega).

### Western Blot Analysis

30 μg amounts of protein and 5× loading buffer was fully mixed and boiled in a constant temperature water bath at 95°C for 5 min to denature the protein. The protein was separated by sodium dodecyl sulfate polyacrylamide gel electrophoresis and transferred to PVDF membranes. The membranes were incubated with primary antibodies overnight at 4°C, horseradish peroxidase-conjugated secondary antibodies for 2 h at room temperature and enhanced chemiluminescence kit (Sigma, United States) according to the manufacturer’s instructions and visualized with a ChemiDoc XRS gel imaging system (Bio-Rad, United States).

### Histopathological Examination

The tumor tissues of three nude mice in each group were randomly taken for histopathological examination. The tumor tissues were conducted fixation, dehydration, transparent, wax dipping, embedding and then cut into 4 µm slices for hematoxylin and eosin (HE) staining, the expression of Ki67, TUNEL and β-catenin according to the manufacturer’s instructions and appropriate dilution ratio. The stained slides visualized under a TCS SP8 microscope (Leica, Germany).

### HCC Xenograft Model

The HepG2 cells grown were digested and centrifuged, resuspended in PBS solution, and 5×10^6^ cells were injected into the right back of each four-week-old male Balb/c nude mouse (Shanghai SLAC Lab. Animal Co., Ltd.). After 2 weeks, when the tumor grew to about 1 cm^3^, mice were treated with 1 mg/kg sempervirine by intraperitoneal injection once daily for 2 weeks. Sorafenib is given orally at 10 and 30 mg/kg, respectively. The growth of tumor and the physical condition of nude mice were observed every day. Animal experiments were approved by the Committee on the Ethics of Animal Experiments of the Fujian Medical University.

### Statistical Analysis

Results were expressed as mean ± SEM from three independent experiments. The results were statistically evaluated using by one-way analysis of variance (ANOVA) followed by the Dunnett’s test. A value of *p* < 0.05 indicates a significant difference.

## Results

### Sempervirine Exhibited Selective Cytotoxicity Toward HCC

**FIGURE 1:**
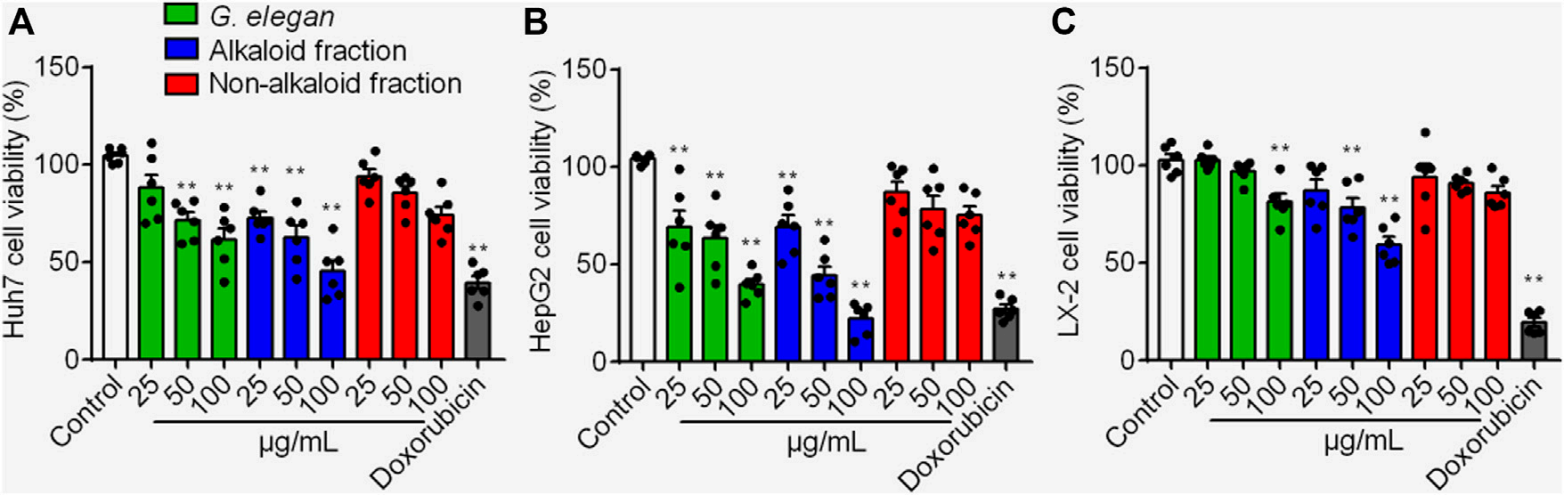
The cytotoxic effects of the alkaloid fraction, non-alkaloid fraction, and whole extracts of *G. elegans* on Huh7 **(A)** HepG2 **(B)** and LX-2 **(C)** cells. Cell viability was assayed for 24 h **p < 0.01 *vs*. Control.

**FIGURE 2:**
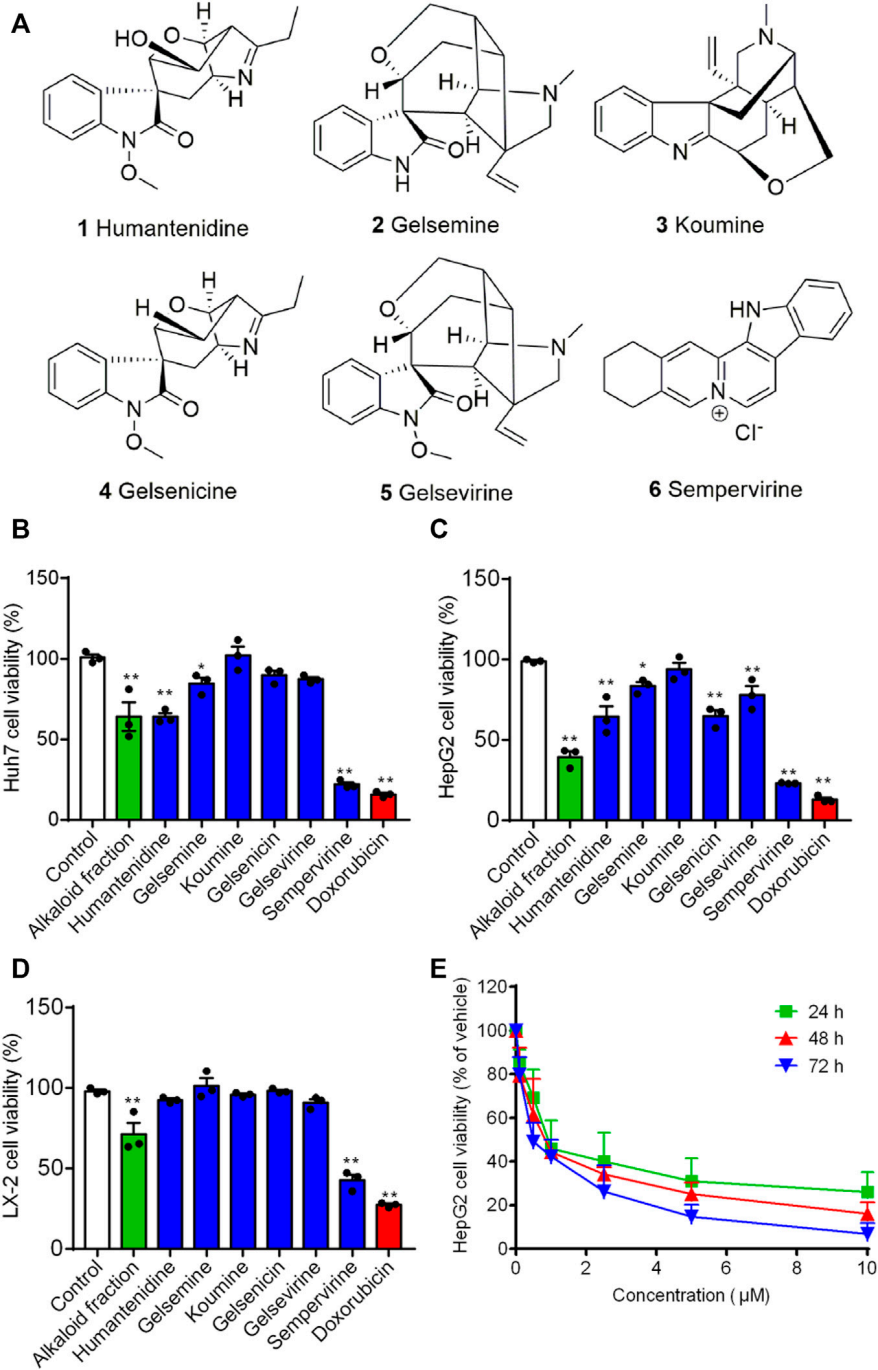
The effect of alkaloids on growth-inhibiting activity in HepG2, Huh7 and LX-2 cells. **(A)** The chemical structure of alkaloids. Huh7 **(B)** HepG2 **(C)** and LX-2 **(D)** cells were treated for 24 h. Cell viability was assayed by CCK8. **(E)** Sempervirine (0, 0.1, 0.5, 1, 2.5, 5, 10 μM) inhibited HepG2 cells growth for 24, 48, and 72 h *p < 0.05; **p < 0.01 *vs*. Control.

HepG2, Huh7 and LX-2 were used to assess the inhibition effects of the alkaloid fraction, non-alkaloid fraction, and whole extracts of *G. elegans* (Figure 1). Furthermore, six alkaloid compounds extracted from the alkaloid fraction of *G. elegans* were evaluated the cytotoxicity *in vitro* (chemical structure shown in Figure 2A). Among the alkaloids examined at 10 μM, sempervirine showed best effects to inhibit the proliferation of Huh7 and HepG2 cells (Figures 2B,C). Interestingly, cytotoxicity analysis showed that sempervirine was found to be relatively low cytotoxic to LX-2 cells (Figure 2D). In addition, the CCK8 results indicated that sempervirine inhibited HCC growth in a dose and time dependent manner (Figure 2E).

### Sempervirine Induced Cell Apoptosis

We next determined whether sempervirine could induce cell apoptosis (Figure 2A). 24 h after the alkaloid fraction (10 and 100 μg/mL) and sempervirine (1 and 10 μM) treatment, HepG2 cells were suffered significantly apoptosis. Next, we analyzed morphological nuclear changes and found that sempervirine significantly causes nuclear contraction and DNA breakage in HCC (Figure 2B). The data indicated that sempervirine induces HepG2 cells apoptosis.

### Sempervirine Induced Cell Cycle Arrest in G1 Phase

**FIGURE 3:**
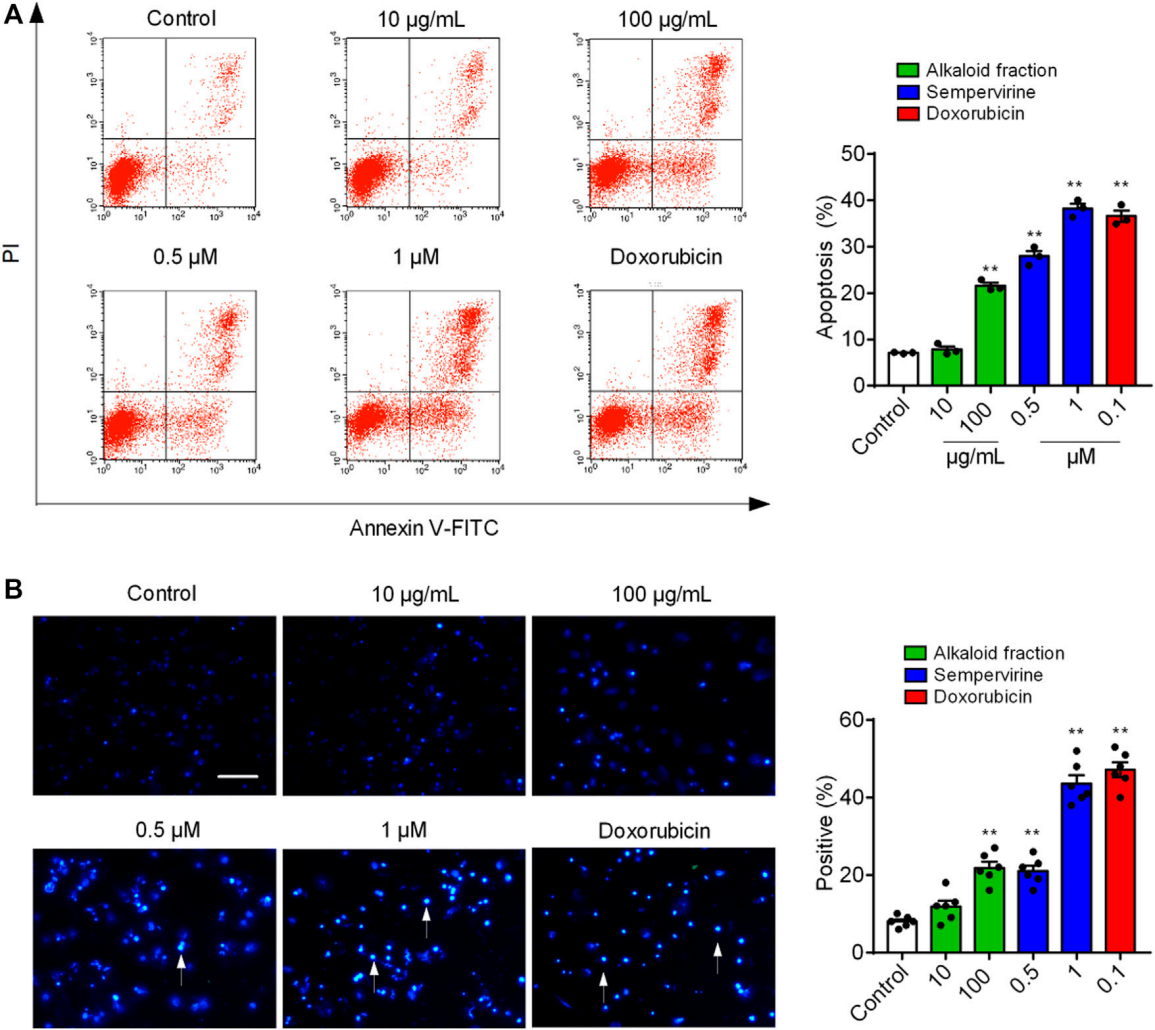
Sempervirine induced apoptosis in HepG2 cells. **(A)** Apoptosis was evaluated by flow cytometry. The *X-* and *Y*-axes represent annexin V-FITC staining and PI, respectively. **(B)** The morphological nuclear changes in HepG2 cells treated with sempervirine at different concentrations. The cells were stained with Hoechst 33258 for 30 min in the dark to examine the cleaved nuclei. **p < 0.01 *vs*. Control.

**FIGURE 4:**
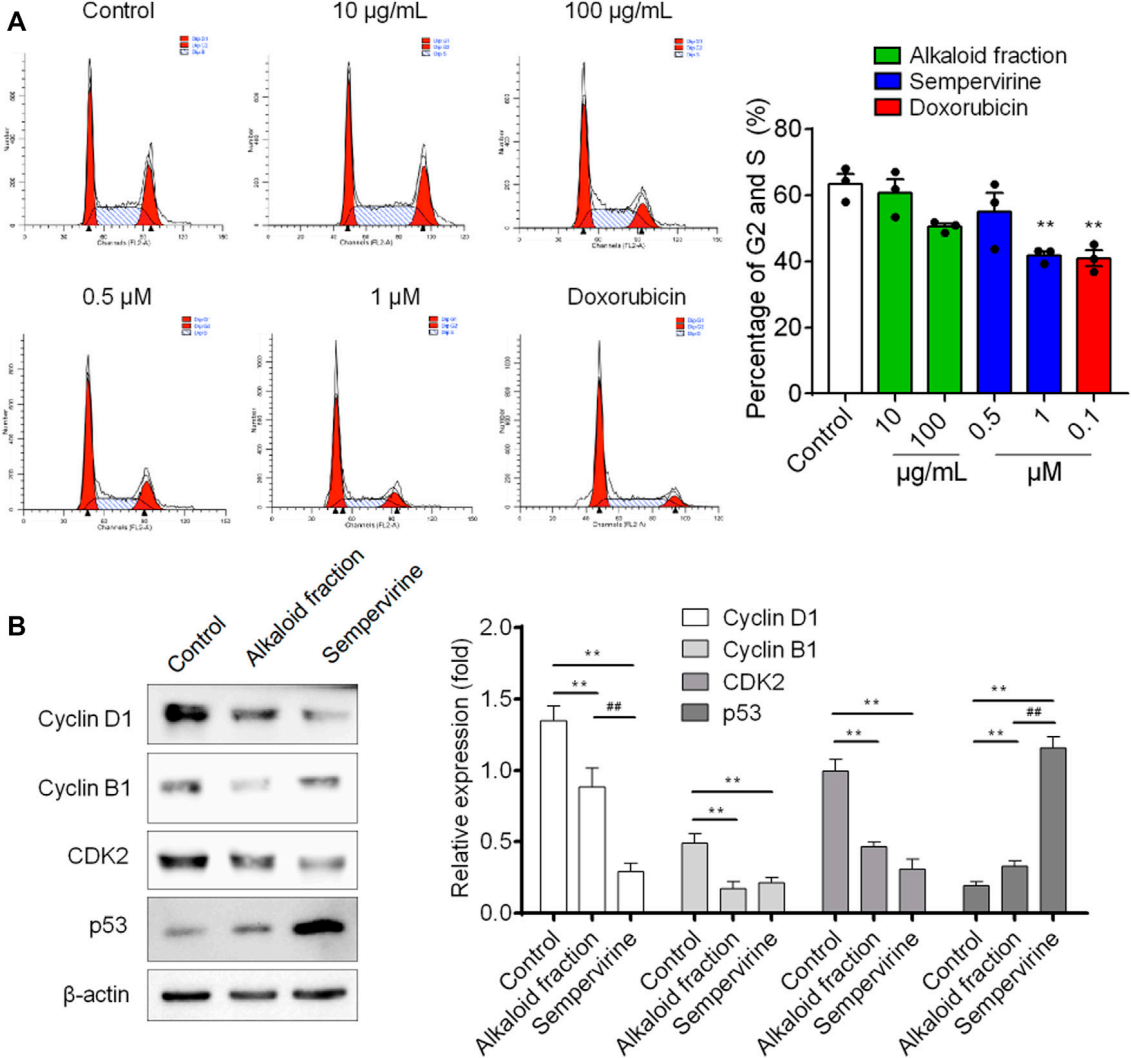
The effect of sempervirine on the cell cycle and the expression of cell cycle regulators in HepG2 cells. **(A)** Sempervirine treatment induced a dose dependent increase in the proportion of cells in the G1 phase and a decrease in cells in the S and G2 phases compared to the control. **(B)** Representative pictures for p53, CDK2, cyclin B1 and cyclin D1 protein expression by western blot analysis. **p < 0.01 *vs*. Control, ^##^ *p* < 0.01 *vs*. the alkaloid fraction.

Flow cytometry was used to analyze the DNA content. Sempervirine induced a dose-dependent increase in the proportion of G_1_ phase and decrease in the S and G_2_ phases (Figure 3A). Furthermore, the expression levels of p53 was markedly increased, whereas cyclin D1, cyclin B1 and CDK2 expression were significantly decreased after sempervirine treatment (Figure 4B). These results revealed that sempervirine induced p53 activation and arrested cell cycle in G_1_ phase.

### Sempervirine Inhibited HCC *In Vivo*

**FIGURE 5:**
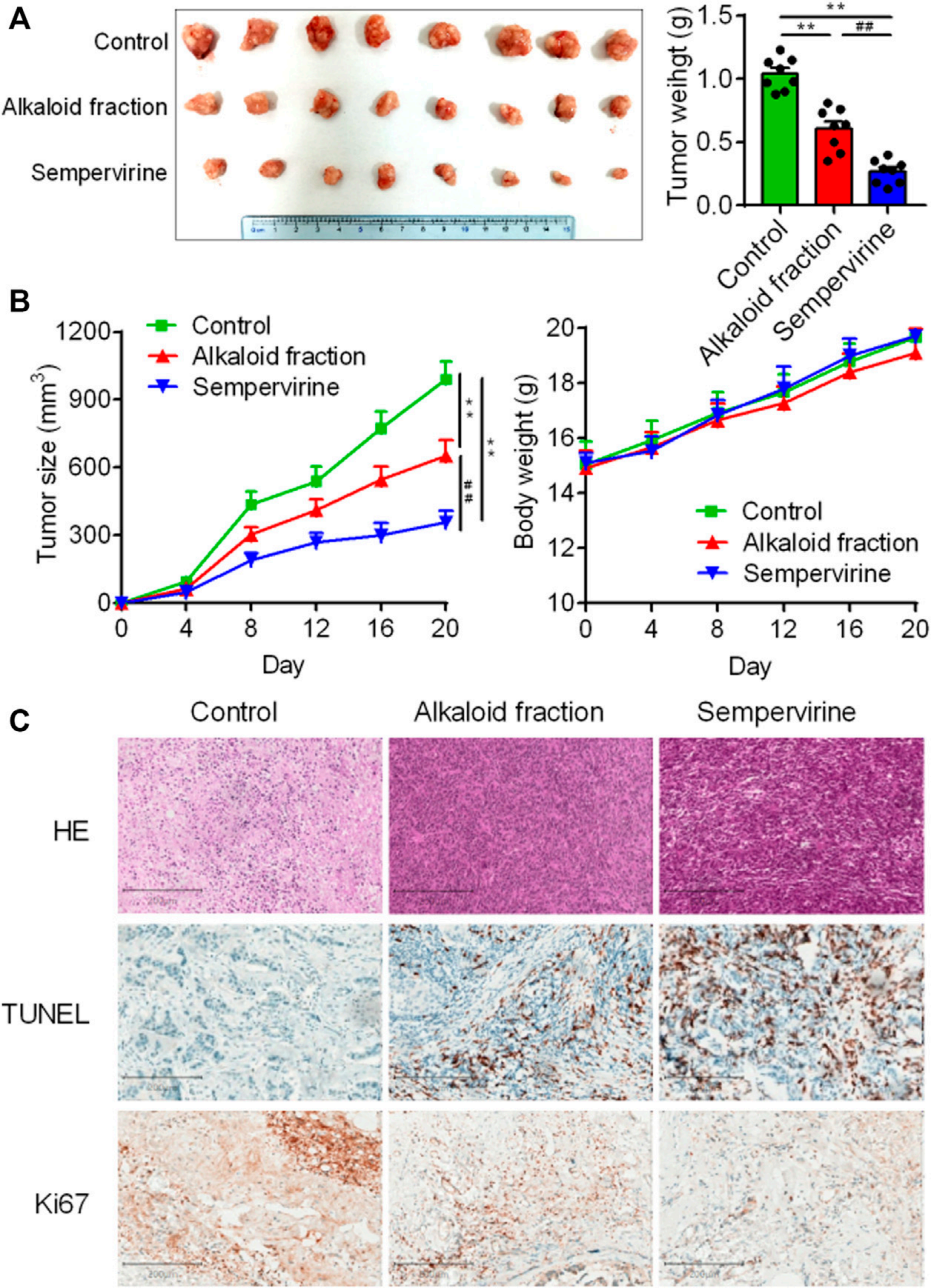
Sempervirine inhibited HCC *in vivo*. **(A)** Images of HepG2 subcutaneous inoculation tumors in mice intravenously treated with the sempervirine for 2 weeks. Quantification of the volumes of tumors was shown. **(B)** Growth curves of the tumors and quantification of body weights of the mice during treatment. **(C)** Representative HE staining, the proliferation marker Ki67 and the apoptosis marker TUNEL of xenografts treated with the sempervirine and the alkaloid fraction. Scale bars = 200 μm. Data are presented as means ± SEM. **p < 0.01 *vs*. Control, ^##^ *p* < 0.01 *vs*. the alkaloid fraction.

Further *in vivo* results shown that sempervirine treatment significantly inhibited HepG2 tumor growth rate and size (Figure 5A). No body weight loss was observed in sempervirine-treated mice (Figure 5B). In addition, Ki67 and TUNEL assay of xenograft tumor tissues were performed to measure proliferation and apoptosis of HepG2 cells in the xenograft model, the results suggested that sempervirine significantly inhibit cell proliferation and induce apoptosis (Figure 5C). These results indicated that sempervirine is a potential therapeutic agent for HCC *in vivo*.

### Sempervirine Enhanced the Anti-tumor Effects of Sorafenib

**FIGURE 6:**
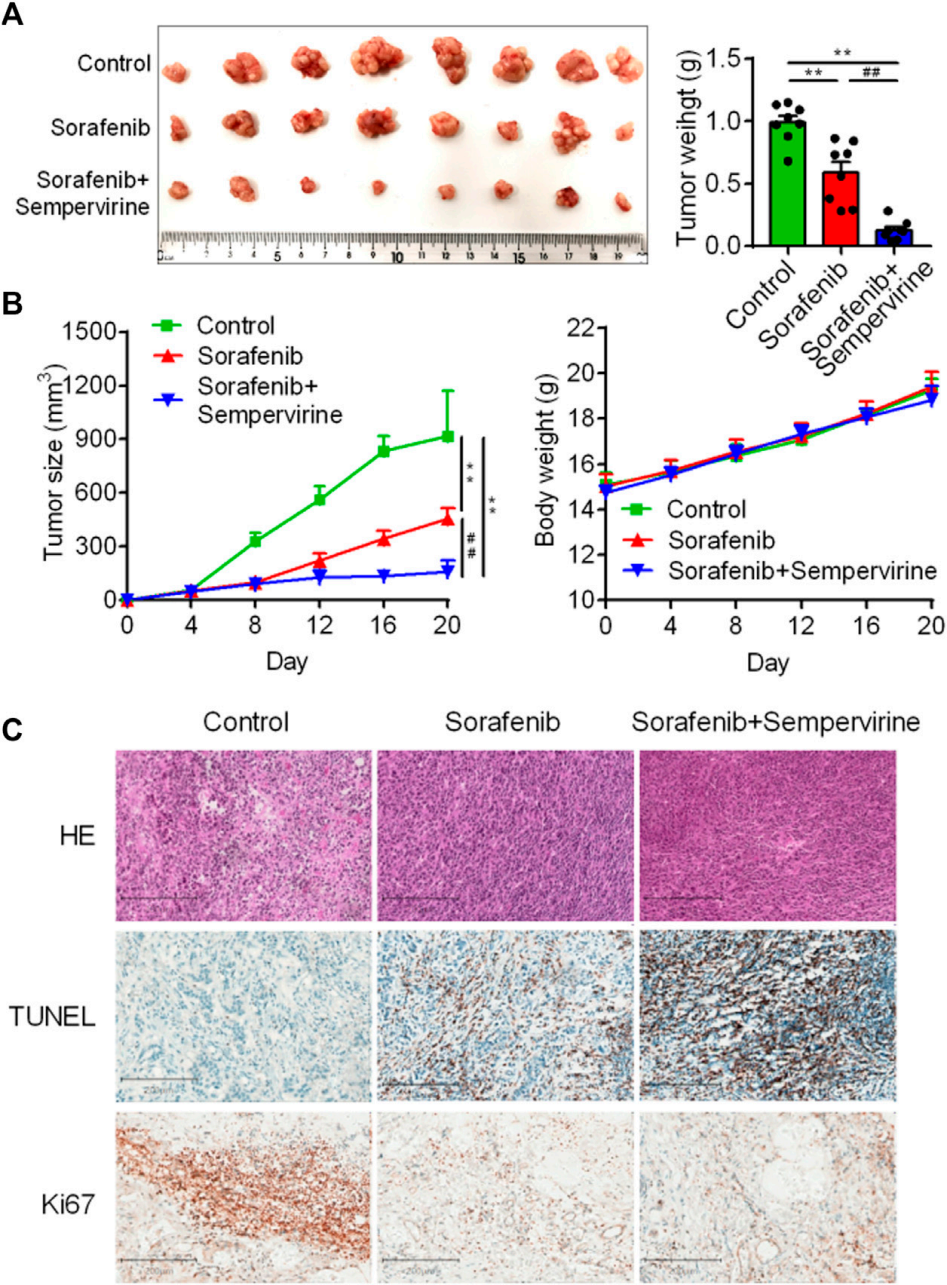
Sempervirine enhanced anti-tumor activity of sorafenib. **(A)** Representative bioluminescence images of HepG2 hepatic tumors in mice treated with sempervirine and sorafenib for 2 weeks. **(B)** Quantification of tumor volumes and tumor growth curves in mice. **(C)** Representative HE staining, Ki67 and TUNE of xenografts treated with the sempervirine and the combination group. Scale bars = 200 μm. Data are presented as means ± SEM. **p < 0.01 *vs*. Control, ^##^ *p* < 0.01 *vs*. the combination.

Sorafenib is a clinically first-line drug for advanced HCC, with limited curative effect and easy to develop drug resistance. Therefore, the synergist of sorafenib is also one of the hotspots in the development of HCC drugs. The results showed that the effect of the combination of sorafenib (10 mg/kg) and sempervirine was more excellent to that of sorafenib at high dose (30 mg/kg) (Figures 6A,B). HE staining showed that the combination of sorafenib and BD and sorafenib high dose treatment could significantly induce tumor tissue necrosis, TUNEL showed that the combination group and sorafenib high dose group could significantly induce the apoptosis of hepatoma tumor cells, and Ki67 showed that the combination group and sorafenib high dose group could significantly inhibit the proliferation of hepatoma tumor cells (Figure 6C). These findings proved that sempervirine possessed synergistic effect with sorafenib.

### Sempervirine Inhibited Wnt/β-Catenin Pathway and Induced Apoptosis *In Vivo*

**FIGURE 7:**
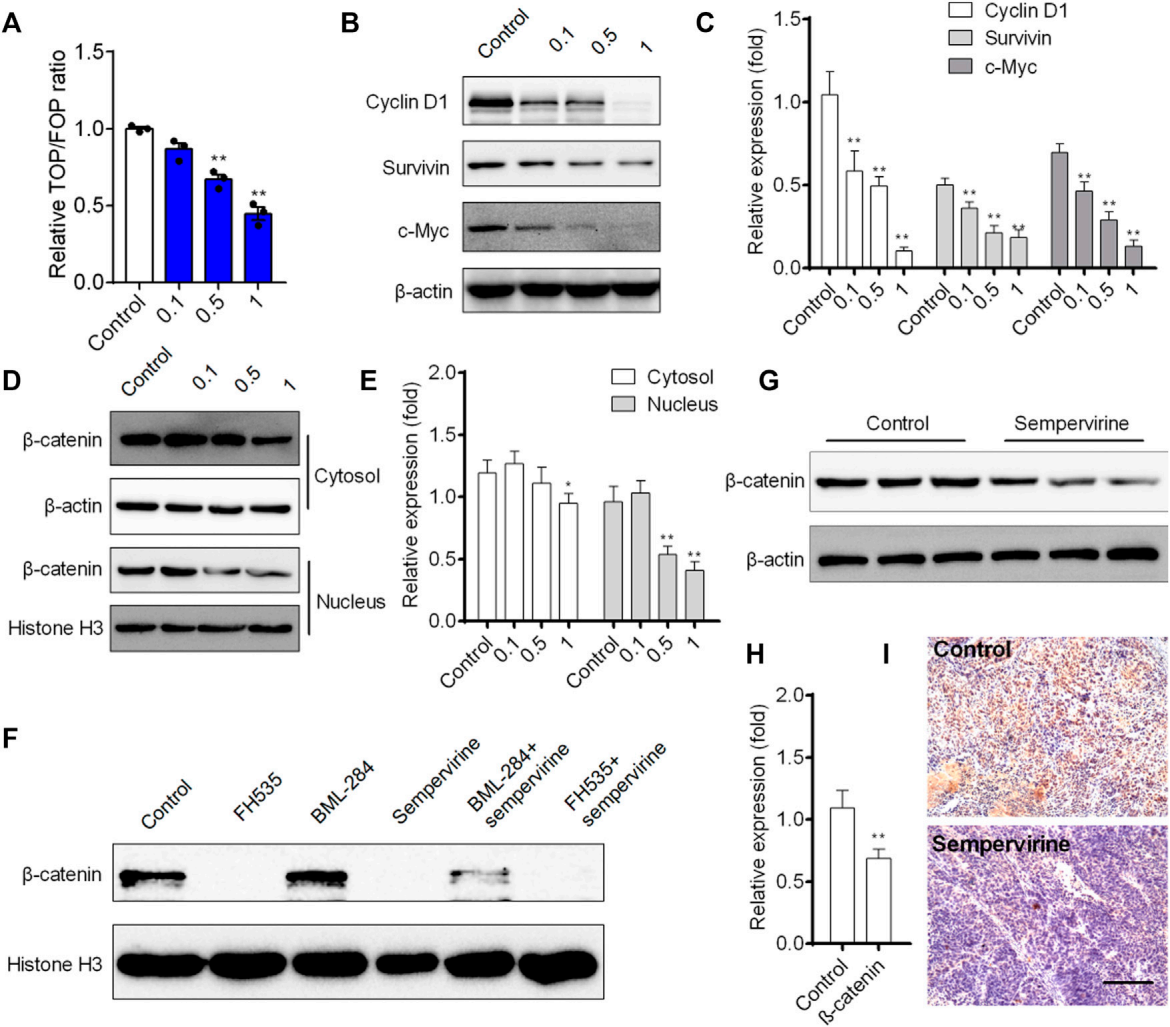
Sempervirine inhibited Wnt/β-catenin pathway and induced apoptosis *in vivo*. **(A)** Cells were treated with different concentrations of sempervirine for 24 h to TOPflash assay. **(B, C)** Western blotting analysis of Wnt/β-catenin target genes survivin, cyclin D1, and c-Myc in HepG2 cells after treated with different concentrations of sempervirine. **(D, E)** HepG2 cells were pretreated with sempervirine for 24 h and the fractioned lysates were analyzed by Western blotting. **(F)** HepG2 cells treated with 10 μM Wnt activator BML-284 or 10 μM Wnt inhibitor FH535 for 24 h in the presence of sempervirine were analyzed by western blots. **(G–I)** The effect of sempervirine on the expression of β-catenin protein *in vivo*. Scale bars = 100 μm. Data are presented as means ± SEM. *p < 0.05; **p < 0.01 *vs*. Control.

We further investigated the effects of sempervirine on the transcriptional activity of Wnt/β-catenin pathway in HepG2 cells. Our results showed that sempervirine inhibited transcription of TCF/LEF in HepG2 cells with a dose-dependent manner (Figure 7A). Furthermore, Wnt/β-catenin target genes survivin, cyclin D1, and c-Myc were significantly decreased after different concentrations of sempervirine treatment in HepG2 cells (Figures 7B,C). The results of separation and detection of nuclear protein and cytoplasmic protein showed that sempervirine could significantly inhibit the level of β-catenin in the nucleus (Figures 7D,E). In addition, the effect of Wnt inhibitor FH535 and agonist BML-284 were used to evaluate the regulatory effect of sempervirine on the lever of β-catenin in nucleus. Sempervirine also decreased the lever of β-catenin in BML-284-treated cells, while the amount of β-catenin after combined treatment with sempervirine was the same as that treated with sempervirine alone (Figure 7F). Moreover, sempervirine suppressed the expression of β-catenin in a xenograft model (Figures 7G,H). These results suggest that sempervirine can significantly inhibit the nuclear aggregation level of β-catenin and inhibit the transcription level of Wnt pathway and thus may induce HepG2 cell apoptosis *via* the Wnt/β-catenin pathway.

## Discussion

Primary liver cancer is one of the common malignant tumors in the world. At present, the first-line drugs for advanced liver cancer are still mainly multiple tyrosine kinase receptor inhibitors. Sorafenib is a first-line anti HCC drug and demonstrates broad oral antitumor efficacy given orally at 7.5–60 mg/kg in panel of human tumor xenograft models. Daily oral administration of Sorafenib (30–60 mg/kg) produces complete tumor stasis during treatment in five of the six models (Wilhelm et al., 2004). However, Sorafenib is easy to develop drug resistance. Drug development strategy of advanced liver cancer is mainly to find molecules targeting new pathways or molecules sensitized by sorafenib. Previous reports indicated that *G. elegans* possessed ideal natural compounds for the treatment of cancer (Liang et al., 2013; Que et al., 2021). Sempervirine, an alkaloid isolated from *G. elegans*, have shown anti-cancer activity (Pan et al., 2016; Caggiano et al., 2020). Our data demonstrated that sempervirine inhibited HCC growth in a dose dependent manner. More interestingly, sempervirine could have synergistic effect with sorafenib *in vitro*.

HCC has the characteristics of rapid proliferation and high degree of malignancy and the treatment of HCC is still an enormous problem (Forner et al., 2012). Several apoptosis inducer have been proven to be clinically effective (Ashkenazi et al., 2017). The present CCK8 assays revealed that sempervirine inhibited induce apoptosis activity. In addition, the growth inhibition induced by sempervirine occurs through the G_1_ phase arrest. Moreover, sempervirine could downregulate the expression of cyclin D1, cyclin B1 and CDK2 and increase p53. Consistently, sempervirine has been identified as a potent inhibitor of MDM2 on p53 (Sasiela et al., 2008).

Wnt pathway is closely related to the differentiation and development of liver and mediates the occurrence and development of a variety of liver diseases (Khalaf et al., 2018; Taciak et al., 2018; Wen et al., 2020). Its key transduction factor β-catenin is closely related to the activation of hepatic progenitor cells. In the mouse liver regeneration model, β-catenin could regulate the specific differentiation of hepatic progenitor cells and induce liver regeneration. In a variety of hepatoma cell lines, β-catenin is closely related to the ability of liver tumor regeneration and invasion (He and Tang, 2020). The over activation of Wnt pathway can induce the occurrence of primary liver cancer in mice. In patients with liver cancer, Wnt pathway is the signal pathway with the highest mutation rate except p53 (Trejo-Solis et al., 2021). Wnt pathway inhibitors can not only inhibit the growth of HCC cells, but also reduce the drug resistance of cells and sensitize chemotherapeutic drugs (Vilchez et al., 2016). β-catenin protein is the key transduction factor to start Wnt pathway transcription. In the inactive Wnt pathway, the level of β-catenin is strictly regulated by GSK3β complex, which keeps Wnt activity at a low level. In tumor cells, Wnt pathway mutation or upstream signal activation makes a large amount of β-catenin accumulate and transfer to the nucleus. Thus, excessive activation of Wnt pathway can promote tumor occurrence and development. The present study found that sempervirine could significantly inhibit the nuclear aggregation level of β-catenin, indicating that sempervirine may act on the transcriptional activity of β-catenin to regulate cell proliferation.

In conclusion, the present study maintained that sempervirine inhibited HCC by arresting G_1_ cell cycle and inducing apoptosis. Furthermore, sempervirine inhibited HCC growth *in vivo* and increatingly, possessed a synergistic effect with sorafenib. In addition, sempervirine could significantly inhibit the nuclear aggregation level of β*-*catenin and inhibit the transcription level of Wnt pathway and thus might induce HCC apoptosis *via* the Wnt/β-catenin pathway.

## Data Availability

The original contributions presented in the study are included in the article/Supplementary Material, further inquiries can be directed to the corresponding authors.
