# Supplementary material for: Sempervirine Inhibits Proliferation and Promotes Apoptosis by Regulating Wnt/β-Catenin Pathway in Human Hepatocellular Carcinoma
Source: Front Pharmacol. 2021 Dec 7;12:806091. doi: 10.3389/fphar.2021.806091 (PMC8689006; doi:10.3389/fphar.2021.806091)

Original images of Western blotting for Figure 4B.

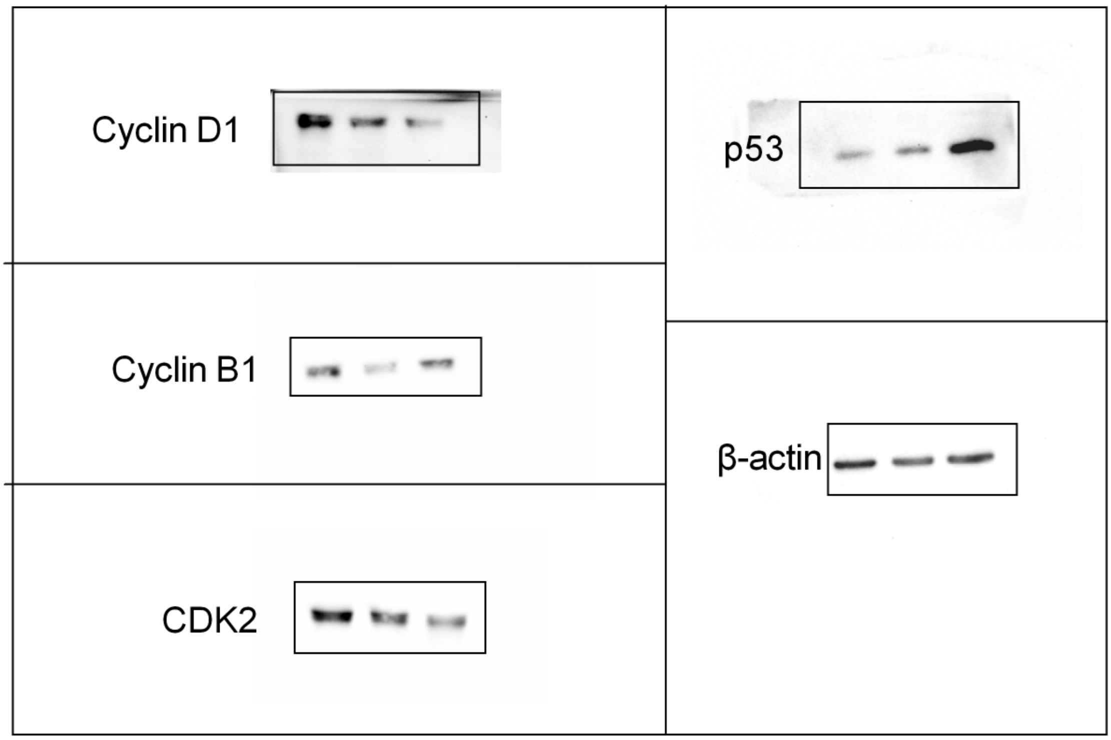

Original images of Western blotting for Figure 7.

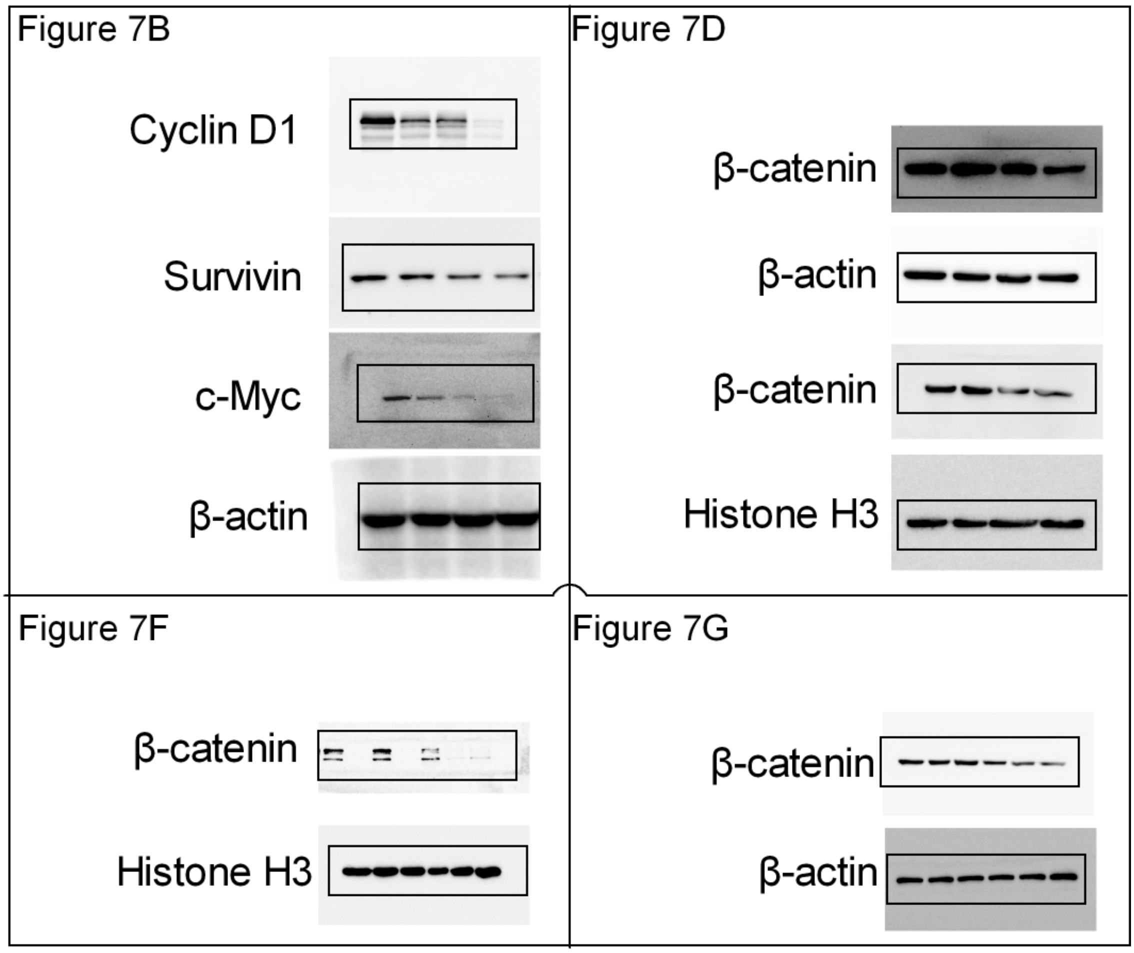

Supplement: Supplementary file 1 [file Image1.pdf]
